# (Near-Infrared) Fluorescence-Guided Surgery Under Ambient Light Conditions: A Next Step to Embedment of the Technology in Clinical Routine

**DOI:** 10.1245/s10434-016-5186-3

**Published:** 2016-03-28

**Authors:** Nynke S. van den Berg, Mitsuharu Miwa, Gijs H. KleinJan, Takayuki Sato, Yoshiki Maeda, Alexander C. J. van Akkooi, Simon Horenblas, Baris Karakullukcu, Fijs W. B. van Leeuwen

**Affiliations:** Interventional Molecular Imaging Laboratory, Department of Radiology, Leiden University Medical Center, Leiden, The Netherlands; Department of Urology, The Netherlands Cancer Institute – Antoni van Leeuwenhoek Hospital, Amsterdam, The Netherlands; Business Incubator, Development Center, Hamamatsu Photonics K.K., Hamamatsu, Japan; Department of Nuclear Medicine, The Netherlands Cancer Institute – Antoni van Leeuwenhoek Hospital, Amsterdam, The Netherlands; Planning and Project Group, Business Planning and Development, Hamamatsu Photonics K.K., Hamamatsu, Japan; Department of Surgical Oncology, The Netherlands Cancer Institute – Antoni van Leeuwenhoek Hospital, Amsterdam, The Netherlands; Department of Head and Neck Surgery and Oncology, The Netherlands Cancer Institute – Antoni van Leeuwenhoek Hospital, Amsterdam, The Netherlands

## Abstract

**Background and Purpose:**

In open surgery procedures, after temporarily dimming the lights in the operation theatre, the Photo Dynamic Eye (PDE) fluorescence camera has, amongst others, been used for fluorescence-guided sentinel node (SN) biopsy procedures. To improve the clinical utility and logistics of fluorescence-guided surgery, we developed and evaluated a prototype modified PDE (m-PDE) fluorescence camera system.

**Methods:**

The m-PDE works under ambient light conditions and includes a white light mode and a pseudo-green-colored fluorescence mode (including a gray-scaled anatomical background). Twenty-seven patients scheduled for SN biopsy for (head and neck) melanoma (*n* = 16), oral cavity (*n* = 6), or penile (*n* = 5) cancer were included. The number and location of SNs were determined following an indocyanine green-^99m^Tc-nanocolloid injection and preoperative imaging. Intraoperatively, fluorescence guidance was used to visualize the SNs. The m-PDE and conventional PDE were compared head-to-head in a phantom study, and in seven patients. In the remaining 20 patients, only the m-PDE was evaluated.

**Results:**

Phantom study: The m-PDE was superior over the conventional PDE, with a detection sensitivity of 1.20 × 10^−11^ M (vs. 3.08 × 10^−9^ M) ICG in human serum albumin. In the head-to-head clinical comparison (*n* = 7), the m-PDE was also superior: (i) SN visualization: 100 versus 81.4 %; (ii) transcutaneous SN visualization: 40.7 versus 22.2 %; and (iii) lymphatic duct visualization: 7.4 versus 0 %. Findings were further underlined in the 20 additionally included patients.

**Conclusion:**

The m-PDE enhanced fluorescence imaging properties compared with its predecessor, and provides a next step towards routine integration of real-time fluorescence guidance in open surgery.

**Electronic supplementary material:**

The online version of this article (doi:10.1245/s10434-016-5186-3) contains supplementary material, which is available to authorized users.

Different groups have reported that for effective intraoperative (near-infrared) fluorescence imaging the lights in the operating room have to be dimmed, or switched off, in order to visualize the fluorescence signal.^1^^,^^2^ This results in temporary stalling of the surgical procedure, even when the fluorescence camera itself is equipped with a white light source.^2^ Therefore, in general, the fluorescence guidance technology is primarily used to provide static confirmatory information regarding the location of lesions.^3^ Ideally, during a surgical procedure the technique would be used to allow the surgeon to excise the lesion of interest under real-time fluorescence guidance.

Previously, in laparoscopic studies using the hybrid tracer indocyanine green (ICG)–^99m^Tc-nanocolloid, we showed that the value of real-time fluorescence guidance significantly increased when the fluorescent signal was displayed within the anatomical context of the patient.^4^ For open surgery procedures, using the Photo Dynamic Eye fluorescence camera (PDE; Hamamatsu Photonics K.K., Hamamatsu, Japan), we saw that in some cases the background signal helped provide anatomical context.^5^–^7^ We reasoned that exploiting this feature further could aid the routine embedment of the technology. Allowing fluorescence guidance under ambient light conditions would, at the same time, help simplify clinical logistics. To achieve our goals, we set out to develop a prototype modified PDE (m-PDE) fluorescence camera, and evaluated it in both a phantom and patient study.

## Materials and Methods

### Fluorescence Camera Systems

We evaluated the newly developed prototype m-PDE fluorescence camera and compared it to the commercially available conventional PDE (c-PDE) fluorescence camera (Hamamatsu Photonics K.K., Hamamatsu, Japan).

The main differences between the c-PDE and the m-PDE are shown in Table 1. Briefly, the light-emitting diode (LED)-based near-infrared excitation light of the c-PDE works in a continuous wave mode, while the illumination source of the m-PDE is pulsed in synchronization with the frame rate of the charge-coupled device (CCD). Here, pulsation means the CCD detector obtains both a fluorescence image containing ambient light background signal and an image of the ambient light background only. Real-time subtraction of the two images then allows the m-PDE to obtain a ‘pure’ fluorescence image (in gray-scale or pseudo-green-color) under ambient light conditions. Second, the m-PDE also allows real-time mixing of the ‘pure’ pseudo-green-colored fluorescence image with the gray-scale anatomical context image. As a third improvement, the m-PDE can also show a white light image in a non-fluorescence imaging setting.

**Table 1:** Characteristics of the conventional and modified PDE fluorescence cameras

	c-PDE	m-PDE
Excitation light source	LED (continuous)	LED (pulsed)
Imaging device	CCD	CCD
Excitation/emission wavelength	760/>820 nm	760/>820 nm
Handheld	Yes	Yes
Pulsed fluorescence imaging	No	Yes
White-light imaging	No	Yes
Focus adjustment	No	Yes
Effective under ambient light conditions	No	Yes
Pseudocoloring	No	Yes (green)
Fluorescence image presented in	Black and white	1. Black and white 2. Pseudocolored green on a gray-scaled anatomical background

*PDE* Photo Dynamic Eye, *LED* light-emitting diode, *CCD* charge-coupled device

### Phantom Study

A 5.0 mg/mL (6.45 × 10^−3^ M) ICG (ICG-Pulsion, 25 mg vial; Pulsion Medical Systems, Munich, Germany)-human serum albumin (HSA; Albuman 200 g/L; Sanquin, Amsterdam, The Netherlands) solution was prepared and diluted 1:1 with HSA in 30 steps down to 9.31 ng/mL (1.20 × 10^−11^ M). From each dilution 100 μL was pipetted in a black 96-well plate (Cellstar; Greiner Bio-One GmbH, Frickenhausen, Germany). The complete dilution range was then evaluated to determine the detection sensitivity of the m-PDE and c-PDE fluorescence camera systems. Hereby, the head of the fluorescence cameras was fixed, perpendicular, at a 14 cm distance from the well-plate surface. This allowed capture of the whole dilution range in the field of view.

Imaging of the plate was performed under different settings—white light (m-PDE only) and fluorescence (both systems) and under various light conditions: (i) all lights in the operating room turned on (halogen satellite lamps directly lighting the sterile field [angle of approximately 45° with regard to the plate surface], the plenum and surrounding lights [both tubular lights]); (ii) satellite lamps directly lighting the sterile field turned off, but the plenum and surrounding lights on (referred to as ‘ambient light’ conditions); and (iii) all lights in the operating room dimmed. For the m-PDE fluorescence camera system evaluation, in all experiments the pseudo-colored green setting was used.

As a reference for the fluorescence intensity measured with the c-PDE and m-PDE fluorescence camera systems, the ICG-based dilution range was also measured on preclinical, cooled, black box, camera systems (IVIS Spectrum, Xenogen Corporation, San Francisco, CA, USA; and the Pearl Impulse, LI-COR Biotechnology GmbH, Hombur, Germany). The fluorescence image obtained with the IVIS Spectrum was presented in a pseudo-colored glow scale, whereas for the Pearl Impulse, the fluorescence signal was presented in a pseudo-colored green scale. For both systems, the fluorescence images were overlaid onto a black and white background image.

For quantification of the fluorescence signal measured with the IVIS Spectrum, in the acquired fluorescence image, regions of interest were drawn surrounding the wells after which Living Image 3D analysis software (version 1.0; Xenogen Corporation) was used to quantify the signal intensity per well.

### Light Spectra Measurements

Light spectra of the different lamps present in the operating room were determined using a Jobin Yvon VS140 linear array fiber spectrometer (Horiba, Kyoto, Japan) in the 300–1200 nm range, with an integration time of 0.1 ms. The fiber was held at a 2-meter distance from the lamp from which the light spectra were measured.

### Absorption and Emission Spectra Measurements of ICG–HSA

The absorption and emission spectrum of ICG-HSA (concentration: 1.5 × 10–9 M) was measured using an Ultrospec 3000 UV/Vis spectrophotometer (Pharmacia Biotech/GE Healthcare Europe GmbH, Eindhoven, The Netherlands) and an LS55 fluorescence spectrometer (PerkinElmer, Groningen, The Netherlands). Solutions were prepared in a 3 mL quartz cuvet (Hellma GmbH & Co. KG, Müllheim, Germany).

### Patient Study

#### Patients

Patients with squamous cell carcinoma of the oral cavity (*n* = 6) or penis (*n* = 5), head and neck melanoma (*n* = 11), or melanoma on the trunk or on an extremity (*n* = 5) scheduled for sentinel node (SN) biopsy with subsequent treatment of the primary tumor/re-excision of the melanoma scar were prospectively enrolled after obtaining written informed consent. All included patients were clinically node-negative as defined by palpation and ultrasound-guided fine needle aspiration cytology. Patient characteristics are shown in Table 2. The study protocol was conducted in accordance with the Helsinki Declaration and approved by the Medical Ethical Committee of the Dutch Cancer Institute – Antoni van Leeuwenhoek Hospital.

**Table 2:** Pre- and intraoperative sentinel node identification findings and pathology results

	Direct camera comparison		Evaluation of m-PDE
	c-PDE	m-PDE	
*Patient characteristics*			
Patients	7		20
Average age, years (range)	64.6 (58–74)		54.4 (34–81)
Male/female ratio	5/2		14/6
Tumor type + tumor stage			
SCC, oral cavity	4		2
T1	4		2
Melanoma (head and neck, trunk, or extremity)	3 (1, 1, 1)		13 (10, 1, 2)
Average Breslow thickness, mm (range)	1.6 (1.2–2.1)		2.1 (0.6–4.0)
Ulceration, yes/no	0/3		3/10
SCC, penis	–		5
T1	–		2
T2	–		3
*Preoperative SN mapping*			
Average injected dose, MBq (range)	69.6 (62.1–77.1)		80.6 (67.3–156)
Preoperative number of SNs identified using SPECT/CT (average, range)	21 (3, 2–5)		51 (2.6, 1–6)
No. of basins (% total), no. of SNs (% total)	16 (100), 21 (100)		40 (100), 51 (100)
Head	1 (6.3), 1 (4.8)		5 (12.5), 6 (11.8)
Auricular	–		–
Parotid gland	–		2 (5.0), 3 (5.9)
Neck (level I–V)	11 (68.8), 15 (71.4)		19 (47.5), 23 (45.1)
Axilla	2 (12.5), 2 (9.5)		2 (5.0), 2 (3.9)
Supraclavicular	–		1 (2.5), 1 (2.0)
Scapular	1 (6.3), 1 (4.8)		–
Groin	1 (6.3), 2 (9.5)		11 (27.5), 16 (31.4)
Average time injection – operation, hrs (range)	5.5 (4.3–6.5)		6.4 (3.5–19.5)
*Intraoperative SN Identification*			
No. of intraoperatively excised SNs (average, range)	27 (3.9, 2–7)		73 (3.7, 1–7)
Radioactive	27		73
Fluorescent	27		73
Blue	1^a^		12^b^
*Specification no. of intraoperative fluorescent SNs (% total) [no. of patients]*			
Visibility through skin	6 (22.2) [2]	11 (40.7) [4]	26 (35.6) [11]
Per basin:			
Head	1	1	1
Auricular	–	–	1
Parotid gland	–	–	2
Neck (level I–V)	5	5	17
Axilla	–	2	2
Supraclavicular	–	–	–
Scapular	–	1	–
Groin	–	2	3
Visibility in vivo (prior to excision)	22 (81.4) [6]	27 (100) [7]	75 (100) [20]
Per basin:			
Head	1	1	5
Auricular	–	–	1
Parotid gland	–	–	8
Neck (level I–V)	19	21	35
Axilla	1	2	2
Supraclavicular	–	–	4
Scapular	1	1	–
Groin	0	2	18
Visibility lymphatic duct	–	2 (7.4) [2]	33 (45.2) [13]
Per basin:			
Head	–	–	2
Auricular	–	–	1
Parotid gland	–	–	4
Neck (level I–V)	–	2	15
Axilla	–	–	2
Supraclavicular	–	–	4
Scapular	–	–	–
Groin	–	–	–
*Pathology*			
No. of tumor-positive SNs (% total)	0/34		4/91 (4.4)
No. of tumor-positive patients (% total)	0/7		4/20 (20.0)

*PDE* Photo Dynamic Eye, *SCC* squamous cell carcinoma, *MBq* mega becquerel, *SN* sentinel node, *SPECT/CT* single photon emission computed tomography combined with computed tomography

^a^In two patients blue dye was used. Here 2 SNs were excised of which 1 was blue at the time of excision

^b^In two patients blue dye was used. Here 2 SNs were excised of which were both blue at the time of excision

### Hybrid Tracer Preparation, Administration, Preoperative Sentinel Node Mapping and (Histo-)Pathology

Preparation and administration of the hybrid tracer ICG–^99m^Tc-nanocolloid, preoperative imaging, and (histo-)pathological specimen analysis for oral cavity cancer,^6^ penile cancer,^8^ and (head and neck) melanoma^7^ have been previously described.

#### Surgical Procedure

**Fig. 1:**
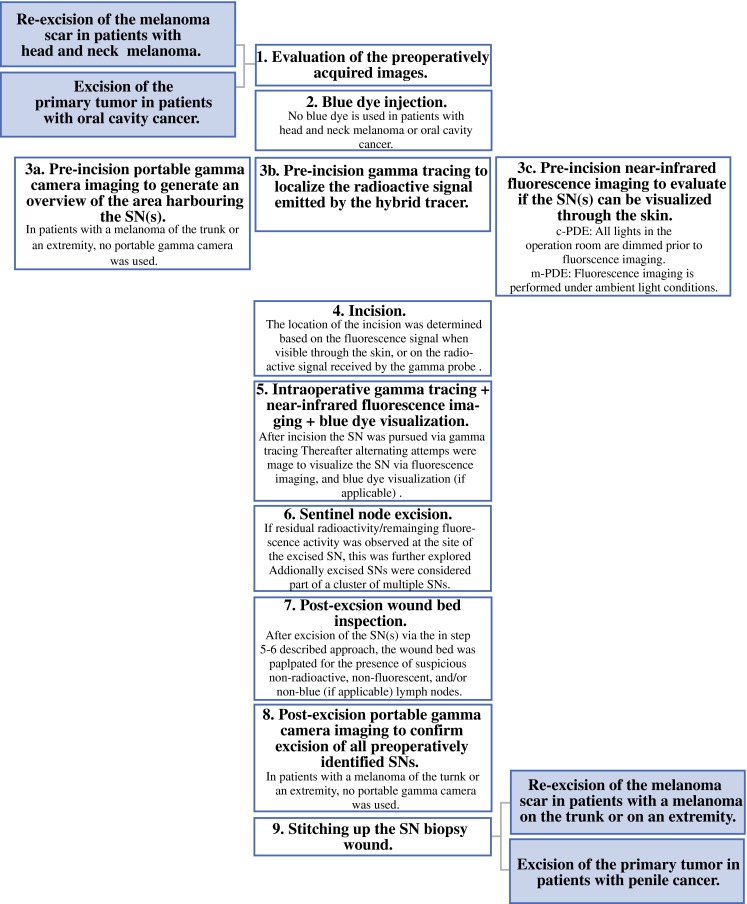
Workflow for sentinel node localization and excision. Following preoperative image analysis by the surgeon to virtually determine the location of the SNs (1), blue dye can be injected (2). Prior to incision a portable gamma camera (Sentinella; Oncovision, Valencia, Spain), a gamma probe (Neoprobe; Johnson & Johnson Medical, Hamburg, Germany), and the fluorescence camera (c-PDE or m-PDE; Hamamatsu Photonics K.K., Hamamatsu, Japan) are use to determine the location of the SNs (3). After incision (4) the SN is pursued via gamma tracing, after which alternating attempts were made to visualize the SN via fluorescence imaging and, when applicable, blue-dye visualization (5). After identification of the SN, the node was excised, after which the wound bed was checked for the presence of residual radioactivity/remaining fluorescence activity at the site of a previously excised SN. Additionally excised nodes were considered part of a cluster of multiple adjacent SNs (6). Following completion of SN biopsy via the combined radio- and fluorescence-guided (and, when applicable, blue dye) approach, the wound-bed was palpated for the presence of suspicious non-radioactive, non-fluorescent and, when applicable, non-blue-dye-stained lymph nodes (8). Thereafter the wound bed was closed (9). *SN* sentinel node, *PDE* Photo Dynamic Eye

 In patients with head and neck malignancies, primary tumor removal or re-excision of the melanoma scar was completed prior to performing SN biopsy. In penile cancer patients and patients with a melanoma on the trunk or on an extremity, SN biopsy was performed prior to treatment of the primary tumor site or the melanoma scar. A schematic overview of the intraoperative SN excision procedure is given in Fig. 1.

## Results

### Phantom Study

#### Reference Fluorescence Data

**Fig. 2:**
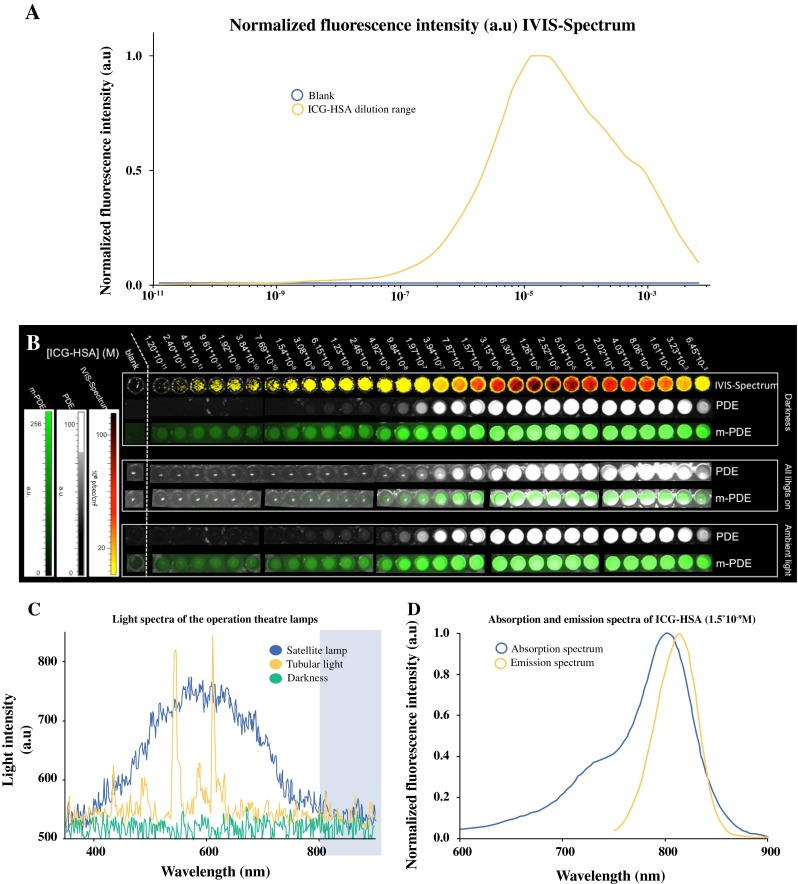
Determination of the sensitivity of the m-PDE and c-PDE fluorescence camera systems for ICG–HSA. (**a**) Fluorescence intensity curve of the various steps of the dilution range measured with the IVIS Spectrum. (**b**) Visual fluorescence images obtained with the IVIS Spectrum, c-PDE, and m-PDE when measured in full darkness, with all lights in the operating room turned on (satellite lamps, plenum, and surrounding lights), and with the satellite lamps directly lighting the sterile field turned off, but the plenum and surrounding lights on. (**c**) Light spectrum of the lamps present in the operating room. The *light blue* area shows the area in which ICG emits its light. (**d**) Absorption and emission spectrum of 1.50 × 10^−9^ M ICG–HSA. *ICG* indocyanine green, *HSA* human serum albumin, *PDE* Photo Dynamic Eye

Figure 2a illustrates the relation between the ICG–HSA concentration and the fluorescence intensity measured with the IVIS Spectrum. Under black-box conditions, the lowest concentration evaluated (1.20 × 10^−11^ M ICG–HSA) could be easily detected using this system (Fig. 2b). The Pearl Impulse showed a similar detection range (data not shown).

Spectral analysis of the light emissions encountered for the different light settings evaluated in the operating room (Fig. 2c) revealed that the light spectrum of the (halogen) satellite lamps gives a broad emission spectrum that shows significant overlap with the spectral area where the ICG emission is collected. The severity with which the satellite lamps influenced ICG detection depended on the angle under which the satellite lamp was placed relative to the phantom. Hereby, the sensitivity for ICG was highest when the satellite lamp was angled so that the reflected satellite lamplight did not align with the position of the fluorescence camera. The normal surrounding lamps (tubular lights) displayed an assembly of light peaks, with the most pronounced emission maxima at 545 and 612 nm, which showed a limited degree of spectral overlap with the emission peak of ICG (Fig. 2c, d).

### Detection Sensitivity Photo Dynamic Eye (PDE) Systems

 Visual inspection of the fluorescence images generated by the m-PDE yielded similar detection sensitivities as reported for the IVIS Spectrum above (1.20 × 10^−11^ M ICG–HSA) (Fig. 2b) when fluorescence imaging was performed in the dark or under ambient light conditions (surrounding lights and plenum turned on) (Fig. 2b). With all the lights turned on, including the satellite lamps, the fluorescence detection sensitivity for the m-PDE system slightly dropped to 2.40 × 10^−11^ M ICG–HSA.

With the c-PDE system, a detection sensitivity of 3.08 × 10^−9^ M ICG–HSA was found under dark conditions (Fig. 2b). This dropped to 4.92 × 10^−8^ M ICG–HSA when all the lights in the operating room were turned on (Fig. 2b). This two-to-three orders of magnitude difference indicates the m-PDE fluorescence camera system can better cope with the background light present in an intraoperative setting.

### Patient Studies

#### Conventional PDE Versus Modified PDE Fluorescence Camera System

In the comparison study in seven patients (oral cavity cancer (*n* = 4) and melanoma (*n* = 3)), a total of 27 SNs were harvested (average 3.9, range 2–7) (Table 2). Initial evaluations performed with the satellite lamps turned on were of limited success and proved to be highly dependent on the positioning of the lamps. For that reason, in this comparison study evaluations were performed with either the satellite lamps dimmed or with these lights turned on, but faced away from the surgical wound bed.

With the m-PDE, under ambient light conditions all SNs evaluated could be easily visualized (100 %). For the c-PDE, with all lights in the operating room dimmed an overall detection rate of 81.4 % was found. The m-PDE system visualized 40.7 % of the SNs transcutaneously (11 SNs, 4 patients; ambient light conditions), while the c-PDE system visualized only 22.2 % (6 SNs, 2 patients; dimmed light conditions). In two patients, a lymphatic duct leading to an SN was visualized with the m-PDE (ambient light conditions), whereas no lymphatic ducts could be visualized with the c-PDE (dimmed light conditions). Further detailed results can be found in Table 2.

Electronic supplementary Fig. SI1 presents the surgical workflow for the c-PDE (Fig. SI1a) and m-PDE (Fig. SI1b) fluorescence camera system. When using the c-PDE (Fig. SI1a), lights in the operating room had to be dimmed in order to visualize the SNs. This temporarily stalled the surgical procedure. Forceps were often placed at the location of the SN, after which the lights in the operating room were turned back on to visually confirm the localization of the SN. This was followed by SN excision and fluorescence imaging to confirm removal of the SN. This process was repeated for each individual SN.

**Fig. 3:**
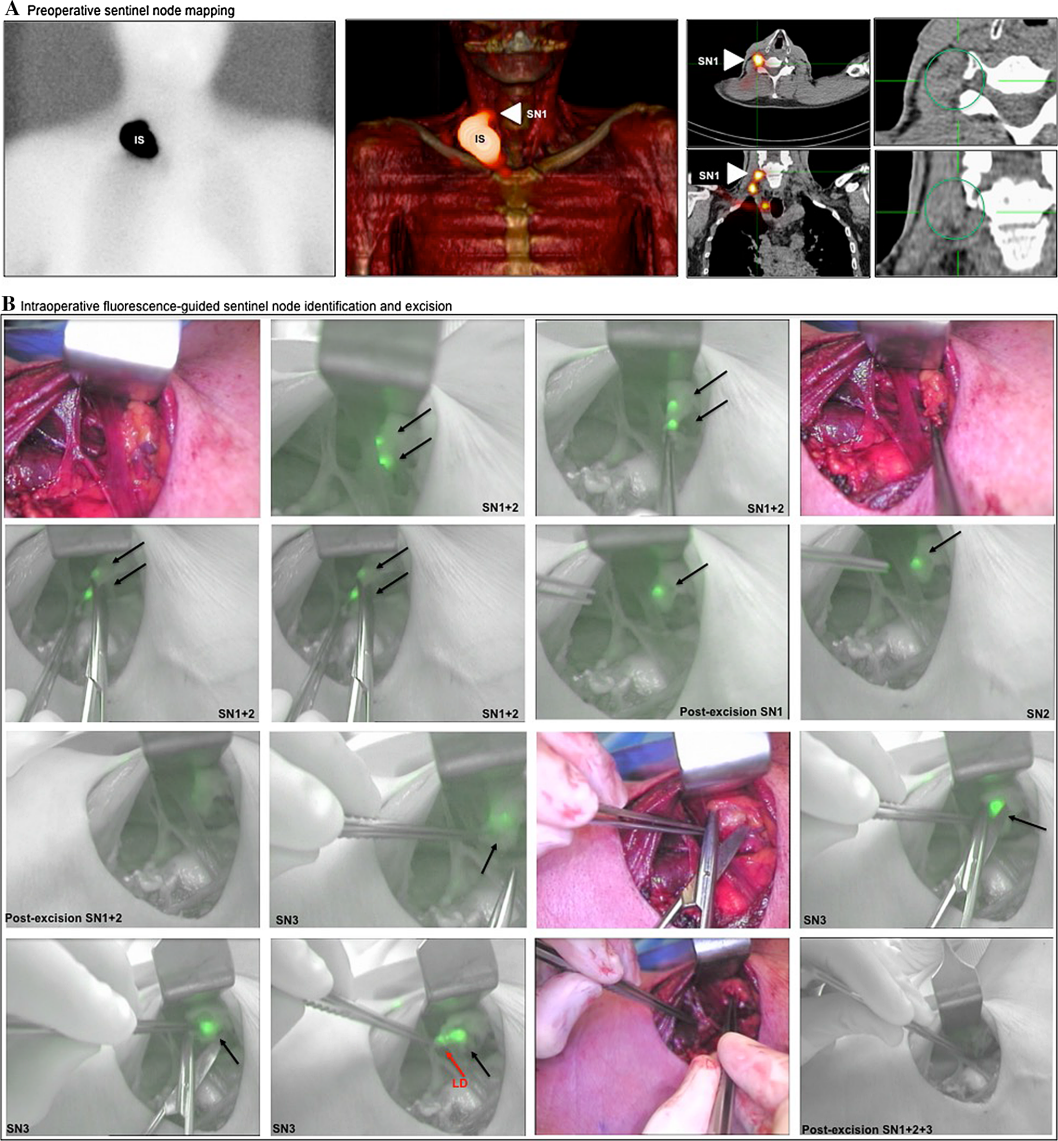
Fluorescence-guided sentinel node excision in a patient with a melanoma of the neck. (**a**) Preoperative imaging. *Left* Static lymphoscintigram acquired 2 h after hybrid tracer injection showing only the IS. *Middle* Following fusion of the acquired SPECT and CT images, a 3D volume rendering was generated showing the injection site, as well as an SN in level IV (*white arrow*) and a supraclavicular SN. *Right* Axial fused SPECT/CT (*left*) and CT (*right*) slice showing the SN in level IV being part of a cluster (indicated because no clear node could be identified on the CT, only a strand of tissue). (**b**) After re-excision of the melanoma scar, the SN cluster in level IV was pursued via fluorescence imaging using the m-PDE fluorescence camera. The timeline shows fluorescence-guided excision of this cluster of SNs. Switching between the fluorescence and white light image allowed the surgeon to work under continuous fluorescence guidance. A total of three fluorescent (and radioactive) SNs were removed from the area where the hotspot was seen on SPECT/CT imaging. *IS* injection site, *SN* sentinel node, *SPECT/CT* single photon emission computed tomography combined with computed tomography, *3D* three-dimensional

 When working with the m-PDE (Fig. SI1b), the presence of ambient light, presentation of the pseudo-colored green fluorescence images on a gray-scaled anatomical background, and the ability to switch the m-PDE to white light mode, combined, allowed the surgeon to directly verify the anatomical location of the SNs and proceed with their excision in a sequential manner. Here, the white light mode allowed us to optimally focus the camera. Please see Fig. 3 for a stepwise illustration on the real-time fluorescence-guided excision of three SNs in a cluster under ambient light conditions. It is interesting to note that even with the increased detection sensitivity of the m-PDE fluorescence camera system, excision of the SNs was not hindered by background signals as a consequence of leakage of tracer from damaged lymphatic ducts (Fig. 3).

#### Extended Clinical Evaluation of Modified PDE Fluorescence Camera System

The m-PDE fluorescence camera was further evaluated in an additional 20 patients: oral cavity (*n* = 2), penile cancer (*n* = 5), and (head and neck) melanoma (*n* = 13). From these patients, 73 SNs were harvested (average 3.7, range 1–7), of which 35.6 % (26 SNs; 11 patients) could be visualized transcutaneously (Table 2). Lymphatic ducts draining from the primary tumor were identified in 13 patients and 33 SNs (45.2 %) (Table 2). Transcutaneous SN visualization, as well as visualization of the lymphatic ducts, was most pronounced in patients with drainage to SNs in the neck (Table 2). Examples of our findings are shown in Fig. 3 and electronic supplementary Figs. SI2 and SI3.

## Discussion

In the current study, we evaluated the effect that technical improvements have on the performance of the fluorescence camera. For this, the ‘new’ prototype m-PDE fluorescence camera was evaluated in relation to the ‘old’ c-PDE. After evaluation in a phantom set-up, its value was defined in patients who were to undergo an SN biopsy procedure for (head and neck) melanoma, oral cavity, or urological malignancies using the hybrid tracer ICG–^99m^Tc-nanocollloid. We have previously reported that this hybrid tracer, in combination with the m-PDE’s predecessor (the c-PDE), allowed superior optical SN visualization compared to blue dye in, for example, patients with vulvar or penile cancer,^8^,^9^ or melanoma^7^ (on average, 60.7 versus 96.5 %, respectively).

The increased sensitivity of the m-PDE compared with the c-PDE, as concluded from the phantom studies, translated nicely in an improved clinical utility of the m-PDE. In a comparative series of seven patients, the reported two-to-three orders of magnitude increase in detection sensitivity resulted in a 14.8 % increase in SN visualization. The value of the m-PDE fluorescence camera system was further underlined in 20 additional patients. With the m-PDE, 35.6 % of the SNs could be visualized transcutaneously and, for 45.2 % of the SNs, lymphatic ducts were visualized. Its utility was further enhanced by (1) the fact that the fluorescence image of the m-PDE is corrected real-time for the influence of ambient light, meaning that the lights in the operating theatre did not have to be dimmed when performing fluorescence imaging; (2) the ability of the m-PDE to show the pseudo-colored green fluorescence image on a gray-scale anatomical background image; and (3) its ability to directly switch between the fluorescence light and white light mode. Given the clear clinical potential of this approach for ICG, which is not a particularly bright dye with a relatively short luminescence lifetime, this concept may, in the future, be successfully expanded to other luminescent tracers that have found their way into the clinic.^10^

The technological evolutions realized in the m-PDE help minimize the disturbance of the clinical workflow and help to transform fluorescence imaging from a confirmatory modality to one that provides real-time ‘on-screen’ guidance during SN excision (as illustrated in Fig. 3 and electronic supplementary Figs. SI1 and SI2). This optimized ‘on-screen’ guidance set-up is comparable to the type of guidance obtained during (fluorescence-guided) laparoscopic surgery.^4^,^11^ However, during open surgery procedures, the small overlap of the ICG light spectrum and the light emitted by the satellite lamp (Fig. 2), in combination with the high intensity of this light source (Fig. 2), still meant that the satellite lamps had to be faced away from the surgical wound bed (or turned off) for optimal guidance. With the upcoming modernized operating rooms, in which halogen satellite lamps are exchanged for LED lamps, this effect will likely become less prominent.

In the current study, we evaluated the m-PDE in combination with ICG–^99m^Tc-nanocollloid, a hybrid tracer that was specifically designed as an SN tracer.^12^,^13^ The specificity of this tracer was further confirmed by the minimal leakage from the lymphatic ducts that we observed with the m-PDE (Fig. 3). When compared with other studies using ‘free’ ICG where such leakage is more common,^11^ this outcome underlines the advantage of using an SN-specific tracer for SN biopsy procedures. From a technical perspective, the advantages the m-PDE has can, in the future, also provide value in applications for which ‘free’ ICG is used, e.g. during angiography applications such as free-flap reconstruction^14^ or partial nephrectomy,^15^ for lymphedema imaging,^16^ lymphatic mapping^11^ or the identification of postoperative lymphatic leaks,^17^ or the for the identification of metastases in the liver.^18^

## Conclusion

The m-PDE fluorescence camera system enhances the fluorescence imaging properties and simplifies the workflow compared with its predecessor. We thus think it provides a critical next step in the routine use of fluorescence-guided surgery.

## Electronic supplementary material

Below is the link to the electronic supplementary material.

Supplementary material 1 (DOCX 2288 kb)
