# Supplementary material for: (Near-Infrared) Fluorescence-Guided Surgery Under Ambient Light Conditions: A Next Step to Embedment of the Technology in Clinical Routine
Source: Ann Surg Oncol. 2016 Mar 28;23:2586–95. doi: 10.1245/s10434-016-5186-3 (PMC4927603; doi:10.1245/s10434-016-5186-3)
Supplement: Supplementary file 1 — Supplementary material 1 (DOCX 2288 kb) [file 10434_2016_5186_MOESM1_ESM.docx]

**SUPLEMENTARY INFORMATION**

**SUPLEMENTARY INFORMATION FIGURES + FIGURE LEGENDS**

**
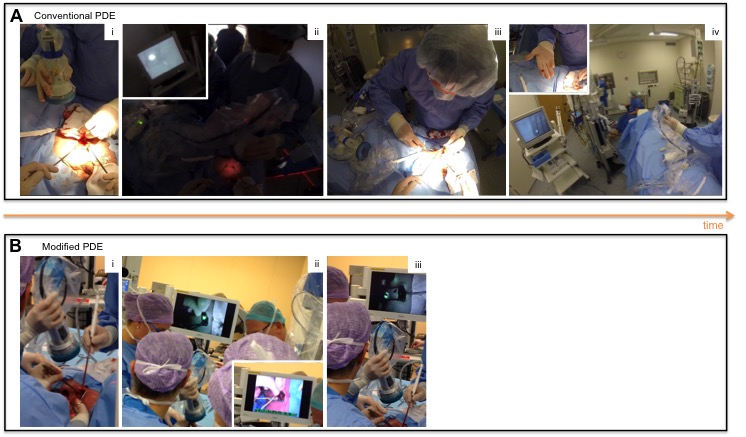
**

**Figure SI1. Operation room logistics for the conventional-PDE and modified-PDE fluorescence camera. A)** Workflow when using the conventional-PDE fluorescence camera: Upon presumed localization of the SN, the camera is brought into position by the operating surgeon (i). Thereafter lights in the operation room are switched off and the surgeon, on-screen, inspects the wound area for the presence of a fluorescence hotspot indicating the SN (ii). A black-and-white fluorescence image is generated by the system (ii, insert). After pinpointing the SN with a forceps, lights in the operation room are turned back on and the SN is excised (iii). Post-excision fluorescence imaging to confirm SN removal (iv; the insert shows the SN lying on the hand of the surgeon). **B)** Workflow when using the modified-PDE fluorescence camera: Upon presumed localization of the SN, the camera is brought into position by the operating surgeon after which the assisting scrub-nurse or fellow will hold the camera to allow for fluorescence-guided SN excision (i). Fluorescence imaging is performed under ambient light conditions. Here the fluorescence signal is displayed on-screen in green on a grey-scaled background. Under real-time fluorescence imaging conditions, the surgeon explores the area harboring the SN (ii; the insert shows the corresponding white light image) and excises it accordingly (iii). SN = sentinel node.

**
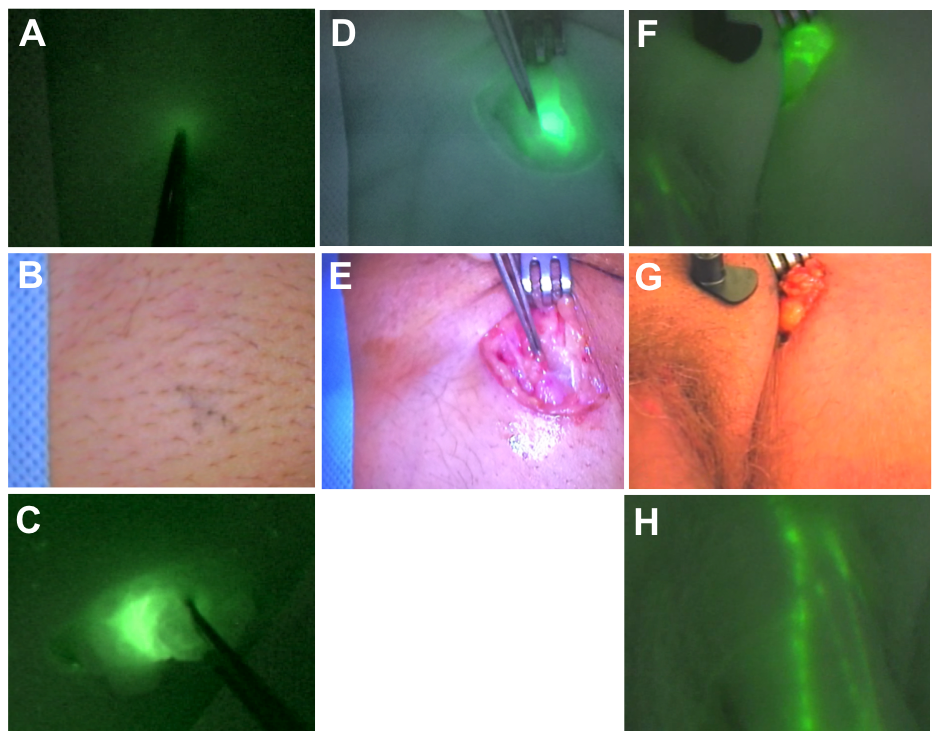
**

**Figure SI2. Examples of the images acquired with the modified-PDE system. A)** Transcutaneous visualization of a SN located in the groin. **B)** Corresponding white light image. **C)** Fluorescence-based SN visualization after the skin was opened. **D)** Intraoperative identification of a non-blue, but radioactive and fluorescent SN in the groin. **E)** Corresponding white light image. **F)** Visualization of the SN in the groin. The left side of the image also shows the lymphatic duct(s) draining to this specific SN. **G)** Fluorescence-based visualization of lymphatic ducts over the penis running to SNs in the groin. **H)** Visualization of lymphatic ducts running over the penis to the SN(s) in the groin. SN = sentinel node; SPECT/CT = single photon emission computed tomography combined with computed tomography.

**
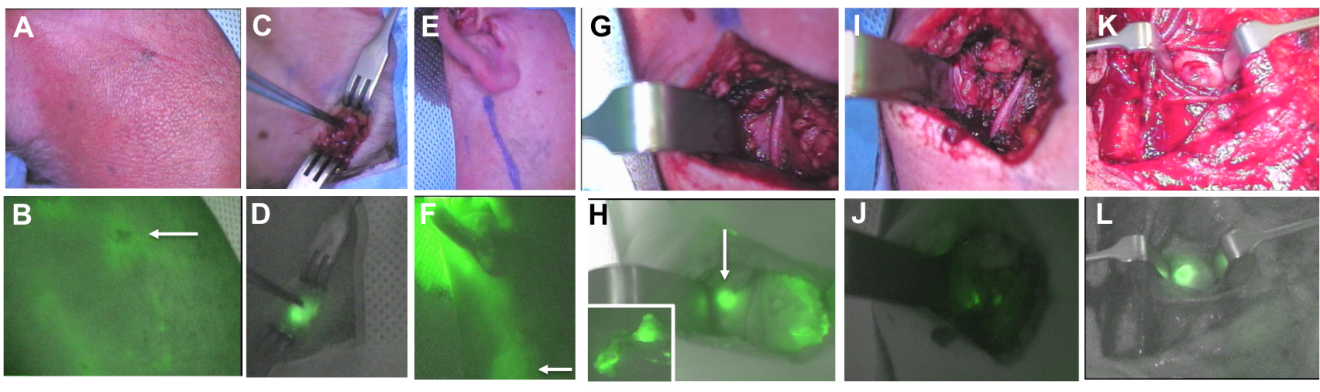
**

**Figure SI3. Examples of the images acquired with the modified-PDE system. A)** Transcutaneous visualization of a suboccipital SN together with the ducts running from the melanoma on the crown of the head to the neck. **B)** Corresponding white light image. **C)** Fluorescence-based visualization of a suboccipital SN. **D)** Corresponding white light image. **E)** Transcutaneous visualization of the lymphatic duct running from the injected melanoma site on the ear to a cluster of SNs in level II of the neck. **F)** Corresponding white light image. **G)** After opening of the skin, a clear fluorescence hotspot could be visualized. During excision here 2 SNs were visualized (insert). **H)** Corresponding white light image. **I)** Post-excision visualization of the remaining lymphatic ducts. **J)** Corresponding white light image. **K)** Fluorescence-based visualization of a deep lying SN in level V in a patient with a melanoma just below the mandibular in the neck. **L)** Corresponding white light image.
